# The Landscape Montage Technique for diagnosing frontotemporal dementia starting as primary progressive aphasia: a case report

**DOI:** 10.1186/s13256-019-2338-7

**Published:** 2020-01-09

**Authors:** Masahiko Takaya, Kazunari Ishii, Isao Kubota, Osamu Shirakawa

**Affiliations:** 1 Department of Neuropsychiatry, Faculty of Medicine, Kindai University, 377-2, Onohigashi, Osakasayama, Osaka, 589-8511 Japan; 2 Department of Radiology, Faculty of Medicine, Kindai University, Osaka, Japan; 3 Department of Rehabilitation, Kindai University Hospital, Osaka, Japan

**Keywords:** Landscape Montage Technique, Logopenic aphasia, Frontotemporal dementia, Primary progressive aphasia, Japanese Standard Language Test of Aphasia

## Abstract

**Background:**

The Landscape Montage Technique was originally developed by Hisao Nakai, a Japanese psychiatrist, to pursue the possibility and application of a psychotherapeutic approach using drawing for patients with schizophrenia. Drawing was initially adopted to evaluate patients with an impaired ability for verbal expression, particularly for the diagnosis and treatment of patients with schizophrenia. Since its development, the Landscape Montage Technique has been utilized in various clinical settings throughout Japan. This study aimed to evaluate the psychiatric conditions of a patient diagnosed as having primary progressive aphasia using the Landscape Montage Technique at a 3-year follow-up.

**Case presentation:**

We present the case of a 64-year-old, right-handed Japanese woman initially diagnosed as having logopenic variant primary progressive aphasia or logopenic aphasia. At a 3-year follow-up, logopenic aphasia progressed to behavioral variant frontotemporal dementia or frontotemporal dementia. According to her husband, she began to have speech difficulties approximately 5 years before her first visit. The results of neurocognitive tests suggested mild cognitive impairment or early stages of dementia. Her clinical dementia rating score was 0.5, suggesting a diagnosis of mild cognitive impairment. She had a Raven’s Colored Progressive Matrices score of 31 out of 36, which indicated a nonverbal cognitive ability that was greater than the 90th percentile for her age. The Japanese Standard Language Test of Aphasia, which was performed at two points during the follow-up, indicated the possibility for a diagnosis of primary progressive aphasia given the progression of her aphasia. Based on her clinical symptoms and Japanese Standard Language Test of Aphasia results, a diagnosis of logopenic variant primary progressive aphasia was established. Magnetic resonance imaging revealed severe predominant left frontal and anterior temporal atrophy, as well as bilateral parietal atrophy. Amyloid beta deposition was negative. At the 3-year follow-up, logopenic variant primary progressive aphasia had progressed to behavioral variant frontotemporal dementia. However, the Landscape Montage Technique allowed for the diagnosis of behavioral variant frontotemporal dementia only 2 years after baseline.

**Conclusions:**

The present study showed that the Landscape Montage Technique can be useful for diagnosing behavioral variant frontotemporal dementia that starts as logopenic variant primary progressive aphasia at earlier stages.

## Background

The Landscape Montage Technique (LMT) was originally developed by Hisao Nakai, a Japanese psychiatrist, to pursue the possibility and application of a psychotherapeutic approach using drawing for patients with schizophrenia [1, 2]. Drawing was initially adopted to evaluate patients with an impaired ability for verbal expression, particularly for the diagnosis and treatment of patients with schizophrenia [1]. Since its origins, LMT has been utilized in various clinical settings throughout Japan [3]. However, to the best of our knowledge, LMT has not been used to evaluate patients with primary progressive aphasia (PPA). In general, most cognitive function tests depend on the subject’s language ability. This is why Japanese Raven’s Coloured Progressive Matrices (RCPM), for example, has been used to evaluate the cognitive ability of patients with aphasia [4]. However, such cognitive tests are not suitable for evaluating the mental state of patients.

PPA is defined as “slowly progressive aphasia” and can be classified into three types: nonfluent/agrammatic variant PPA (nvPPA), semantic variant PPA (svPPA), and logopenic variant PPA (lvPPA) [5]. The imaging characteristics of nvPPA include predominant left posterior front-insular atrophy, hypoperfusion, or hypometabolism; those for svPPA include predominant anterior temporal lobe atrophy, hypoperfusion, or hypometabolism; and those for lvPPA include predominant left posterior perisylvian or parietal atrophy, hypoperfusion, or hypometabolism [5]. The aforementioned PPA pathologies could indicate Alzheimer’s disease, frontotemporal dementia (FTD), or other diseases [5]. The main clinical features of FTD or behavioral variant FTD (bvFTD) include progressive deterioration of behavior and/or cognition [6], whereas the supportive imaging features of bvFTD include frontal and/or anterior temporal atrophy, hypoperfusion, or hypometabolism [6]. These criteria indicate that each type of PPA could have imaging features similar to bvFTD. As such, PPA may possibly progress to FTD and vice versa.

We report a case involving a 64-year-old, right-handed Japanese woman with PPA, which could have been diagnosed as lvPPA or logopenic aphasia [7]. At a 3-year follow-up, lvPPA had progressed to bvFTD [8]. To investigate the progression of the clinical symptoms of lvPPA, LMT was performed longitudinally. To the best of our knowledge, this is the first report suggesting that LMT could be useful for the early diagnosis of bvFTD that starts as PPA.

## Case presentation

We present the case of a 64-year-old, right-handed Japanese woman initially diagnosed as having lvPPA. At a 3-year follow-up, lvPPA had progressed to bvFTD, with results from the Neuropsychiatric Inventory assisting in the diagnosis [8, 9].

Her medical and family history were noncontributory. According to her husband, her speech difficulties began approximately 5 years before her first visit, which he thought was due to psychogenic factors considering that his business had not been doing well during those days. Her husband stated that she had difficulty recalling words [7]. Although his business subsequently improved, her symptoms gradually worsened, which indicated that her symptoms were neurogenic and not psychogenic. She had not received any psychiatric or counseling therapy. Her neurological and mental status examinations were unremarkable. Upon medical interview, her speech was fluent but sparse [7]. Impairment was noted in both “single-word retrieval in spontaneous speech and naming” and “repetition of spontaneous sentence and phrases”, which are important core features of lvPPA (Table 4 in Gorno-Tempini *et al.*, 2011) [5].

**Fig. 1:**
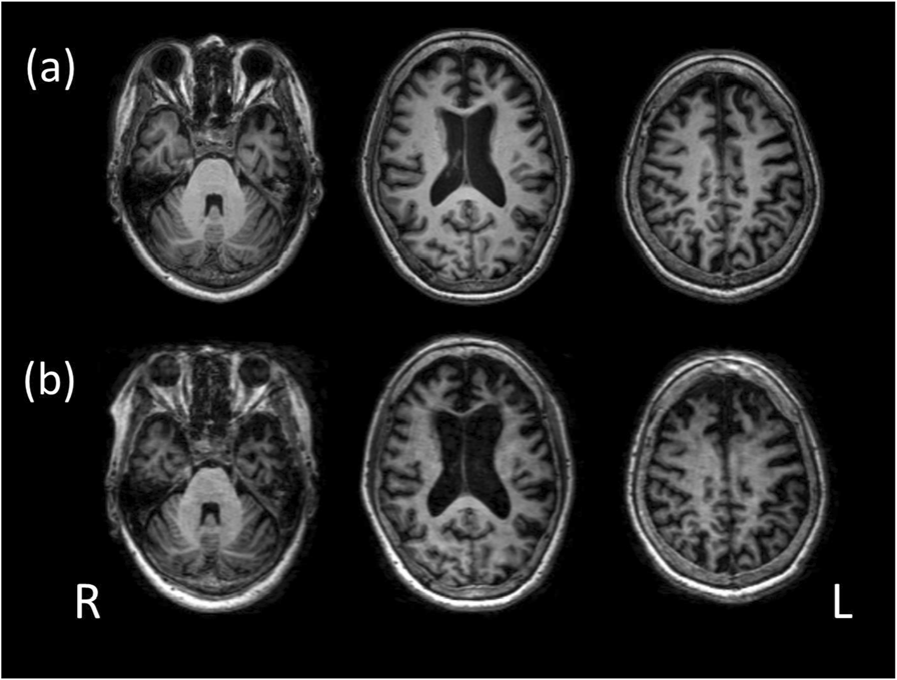
T1-weighed magnetic resonance images showing bilateral frontal and anterior temporal lobe atrophy, as well as bilateral parietal lobe atrophy (**a**). Atrophy progression 30 months later (**b**). **a** has been reprinted with permission from the original publisher (© International Psychogeriatric Association 2017, published by Cambridge University Press) [7]. **b** has been added to the original images described above. *L* left, *R* right

During the first visit, she appeared to recognize what was said. Therefore, cognitive function tests could be performed [7]. Her cognitive ability was assessed using the Mini-Mental State Examination (MMSE), a cognitive subscale of the Alzheimer’s Disease Assessment Scale-Japanese version (ADAS-J cog.), the Clock-Drawing Test, the Frontal Assessment Battery, the Japanese Adult Reading Test, the digit span subtest in the Wechsler Adult Intelligence Scale-Third edition, a logical memory subtest in the Wechsler Memory Scale-Revised, the Rey–Osterrieth Complex Figure Test, and RCPM [7]. The results of these cognitive tests suggested that she suffered from mild cognitive impairment (MCI) or the early stages of dementia. However, she had a clinical dementia rating score of 0.5, which suggested a diagnosis of MCI rather than dementia [10]. Cognitive test results, such as the MMSE and ADAS-J cog, may have underestimated her cognitive function because of aphasia. In fact, her RCPM score was 31 out of 36, which indicated a nonverbal cognitive ability greater than the 90th percentile for her age [4]. Results for the Japanese Standard Language Test of Aphasia (SLTA) indicated the possibility for a diagnosis of PPA given the progression of her aphasia [11] (Table 1). Considering that the SLTA was established during predecessor conferences of the Japan Society for Higher Brain Dysfunction, the primary reference cannot be cited. However, test kits for SLTA are purchasable without difficulty and are prevalent throughout Japan. The process whereby lvPPA was diagnosed can be described by the following criteria (Table 4 in Gorno-Tempini *et al.*, 2011) [5]: observable “Impaired single-word retrieval in spontaneous speech and naming” during the medical interview; subtest 9 of SLTA indicating “Impaired repetition of sentences and phrases” (Table 4 in Gorno-Tempini *et al.*, 2011) [5]; subtest 1 of SLTA indicating “spared single-word comprehension and object knowledge” (Table 4 in Gorno-Tempini *et al.*, 2011) [5]; and no frank agrammatism observed in performing subtest 7 of SLTA as well as in medical interviews, which indicates “Absence of the frank agrammatism” (Table 4 in Gorno-Tempini *et al.*, 2011) [5]. The aforementioned findings satisfied the criteria for “Clinical diagnosis of logopenic variant PPA” (Table 4 in Gorno-Tempini *et al.*, 2011) [5]. Moreover, our patient’s neuroimaging results satisfied the criteria for “Imaging-supported logopenic variant diagnosis” (Table 4 in Gorno-Tempini *et al*., 2011) as shown by magnetic resonance (MR) images revealing predominant left frontal and anterior temporal atrophy at baseline and bilateral parietal atrophy, although parietal atrophy was lesser compared to frontal and temporal atrophy (Fig. 1a) [5]. Thus, she was diagnosed as having lvPPA according to the aforementioned criteria (Table 4 in Gorno-Tempini *et al.*, 2011) [5].

**Table 1:** The results of all subtests of Japanese Standard Language Test of Aphasia

Subtest	Baseline	12 months later
(1) Auditory comprehension of words (to point at pictures) (out of 10)	10	10
(2) Auditory comprehension of short sentences (to point at pictures) (out of 10)	9	9
(3) Auditory comprehension (to obey verbal commands) (out of 10)	7	4
(4) Auditory comprehension (to point at Kana letters) (out of 10)	10	9
(5) Naming (out of 20)	15	6
(6) Word repetition (out of 10)	10	10
(7) Verbal explanation of behavior of persons and movement of others in pictures (out of 10)	8	4
(8) Verbal explanation of Manga (one to six stages)	3	1
(9) Sentence repetition (out of 5)	3	3
(10) Verbal fluency (number of words)	4	1
(11) Reading aloud Kanji words (out of 5)	5	4
(12) Reading aloud Kana letters (out of 10)	10	9
(13) Reading aloud Kana words (out of 5)	5	5
(14) Reading aloud short sentences (out of 5)	4	4
(15) Reading comprehension of Kanji words (to point at pictures) (out of 10)	10	10
(16) Reading comprehension of Kana words (to point at pictures) (out of 10)	10	10
(17) Reading comprehension of short sentences (to point at pictures) (out of 10)	8	8
(18) Reading comprehension (to obey written commands) (out of 10)	5	2
(19) Writing words with Kanji (to represent pictures) (out of 5)	4	4
(20) Writing words with Kana (to represent pictures) (out of 5)	3	1
(21) Writing explanation of Manga (one to six stages)	3	1
(22) Dictation of Kana letters (out of 10)	10	8
(23) Dictation of Kanji words (out of 5)	4	4
(24) Dictation of Kana words (out of 5)	5	1
(25) Dictation of short sentences (out of 5)	0	0
(26) Four arithmetic operations on paper (out of 20)	11	8

This Table is reprinted with permission from the original publisher (© International Psychogeriatric Association 2017, published by Cambridge University Press) [7]

At baseline and 30 months later, our patient’s findings satisfied the criteria for imaging-supported lvPPA diagnosis [5]. However, her imaging results were consistent with bvFTD [5, 6, 8]. Bilateral frontal and anterior temporal lobe atrophy, as well as bilateral parietal lobe atrophy progressed (Fig. 1). Although her clinical symptoms were not consistent with bvFTD at baseline, they were so 3 years later [8] as described by the following subtest (agitation/aggression, apathy, aberrant motor behavior, and eating behavior abnormalities) results (frequency × severity) for Neuropsychiatric Inventory 3 years after baseline: 4 × 2, 4 × 3, 3 × 3, and 4 × 2, respectively [8]. These results satisfied the criteria for probable bvFTD described in Table 3 of Rascovsky *et al.* (2011) [6]. In addition, Pittsburgh Compound B positron emission tomography amyloid images showed negative amyloid deposition in the cerebral cortices, which was described in our previous reports [7, 8]. Moreover, her medical history satisfied other exclusion criteria for bvFTD described in Table 3 of Rascovsky *et al.* (2011) [6]. Therefore, a diagnosis of bvFTD had been established 3 years after baseline [8].

LMT has been longitudinally performed to evaluate the clinical features of patients in a nonverbal manner. Since its development, LMT has been utilized in various clinical settings across Japan. The basic procedure for LMT is as follows. First, the therapist draws a border with a black felt-tip pen on a white A4-sized sheet of paper in the presence of the patient. The therapist then tells the patient, “Now, I would like you to draw a landscape” and “I do not aim to evaluate your drawing ability.” The client is then asked to draw ten items in a predetermined order. The items are described as follows: “a river or rivers,” “a mountain or mountains,” “rice paddy,” “a road or roads,” “a house or houses,” “a tree or trees,” “a person or persons,” “a flower or flowers,” “a creature or creatures,” and “a stone or stones.” Finally, the therapist asks the client to color the images [1–3].

**Fig. 2:**
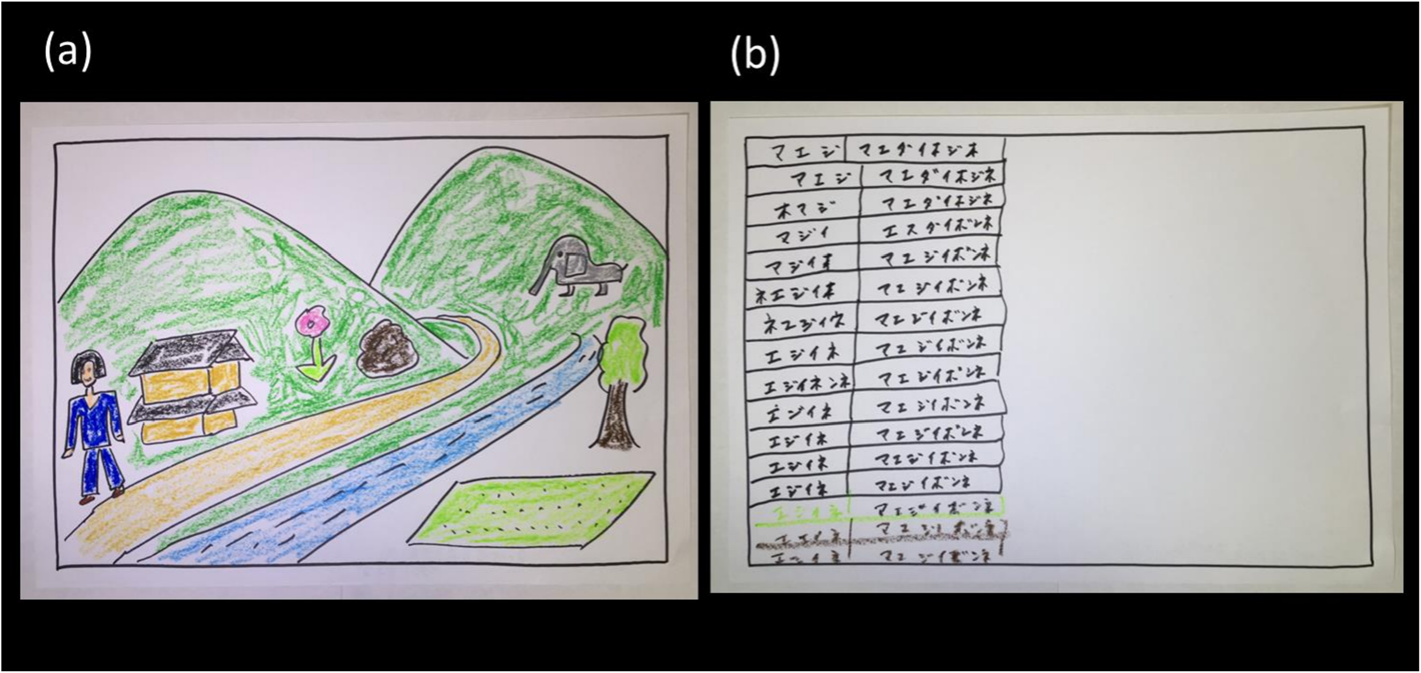
**a** A drawing created at baseline using the Landscape Montage Technique. **b** A drawing created 2 years after baseline using the Landscape Montage Technique

In the present case, LMT was performed on our patient with logopenic aphasia to reveal her clinical features, both at baseline and 2 years later. From baseline until 2 years later, her speech, which stopped after a sentence, was fluent but sparse. She barely managed to answer closed questions 2 years after baseline. Although her listening comprehension and nonverbal expression at the 2-year follow-up seemed similar to those at baseline, LMT results indicated otherwise. The drawing she created at baseline is shown in Fig. 2a; the drawing she created 2 years after baseline is shown in Fig. 2b. The contrast between the two drawings is striking. The former drawing (Fig. 2a) is easy to look at, whereas the latter (Fig. 2b) is confusing. First, Fig. 2b did not seem to depict a “landscape”, nor was each item (for example, “a river or rivers”) drawn at all. Second, in Fig. 2b, she seemed unable to follow instructions, although the drawn items looked similar, which continued until the end of LMT. Each row, or each item, was drawn from top to bottom of the paper. More than 10 items were drawn given that she continued drawing similar stereotyped items irrespective of instructions. She was subsequently diagnosed as having bvFTD, more precisely “probable bvFTD,” 3 years after baseline [6, 8]. However, the drawing in Fig. 2b indicated that she could have already been diagnosed as having “probable bvFTD” only 2 years after baseline [6]. The drawing process revealed one criterion for “possible bvFTD,” namely “simple repetitive movements” (Table 3 of Rascovsky *et al.*, 2011), which could be called “clonic perseveration” [6]. Moreover, her “apathy” and “loss of sympathy or empathy” in the criteria for possible bvFTD (Table 3 of Rascovsky *et al*., 2011) were obvious from observation, thereby satisfying the criteria for possible bvFTD [6]. Both the clinical features and MR images in Fig. 1b satisfied the criteria for “probable bvFTD” (Table 3 of Rascovsky *et al*., 2011) [6].

The timeline has already been presented in Table S1 of our previous report [8]. To outline her medical history, the timeline is again shown as follows: (a) T − 5 years, initial development of symptoms; (b) T, presentation and diagnosis of lvPPA; and (c) T + 3 years, diagnosis of bvFTD without LMT. Hence, our results showed that LMT utilization allowed for the diagnosis of bvFTD at T + 2 years or 1 year before bvFTD was diagnosed without LMT.

## Discussion and conclusion

This case demonstrates the potential of LMT for diagnosing bvFTD at an early stage, particularly among patients whose bvFTD started as lvPPA or other types.

FTD can be diagnosed according to established criteria, which are now predominant and include both observational and neuroimaging findings [6]. Usually, the patients’ family members, caregivers, and attending physicians observe the patients’ behaviors and notice abnormalities therein, which leads to a diagnosis based on criteria. Moreover, these observations can be performed in daily life and upon consultation.

However, LMT can induce abnormal behaviors among patients with bvFTD. This is why the current patient had been diagnosed as having bvFTD a year earlier than usual. This striking discovery regarding the utility of LMT could help in the early diagnosis of bvFTD and the establishment of a care plan.

Some limitations of the present report need to be considered. First, the current report discusses only one case. Therefore, future studies should investigate more cases, particularly other types of PPA. Second, although SLTA was used to evaluate aphasia, this test has been made especially for Japanese-speaking individuals. However, other language versions of SLTA remain to be established and validated in the future. Third, the current report could not demonstrate the utility of LMT for treatment, which should be investigated in the future. Lastly, instructions and procedures for LMT in languages other than Japanese have been quite limited. However, this could change following worldwide spread of LMT in the future.

## Data Availability

Data sharing is not applicable for this article given that no datasets were generated or analyzed during the course of current study.

## Abbreviations

ADAS-J cog: A cognitive subscale of the Alzheimer’s Disease Assessment Scale-Japanese version
bvFTD: Behavioral variant frontotemporal dementia
FTD: Frontotemporal dementia
LMT: Landscape Montage Technique
lvPPA: Logopenic variant primary progressive aphasia
MCI: Mild cognitive impairment
MMSE: Mini-Mental State Examination
MR: Magnetic resonance
nvPPA: Nonfluent/agrammatic variant primary progressive aphasia
PPA: Primary progressive aphasia
RCPM: Japanese Raven’s Coloured Progressive Matrices
SLTA: Japanese Standard Language Test of Aphasia
svPPA: Semantic variant primary progressive aphasia
